# Delays in seeking and reaching care for injured patients in four low-income and middle-income countries: a cohort study

**DOI:** 10.1136/bmjgh-2025-021659

**Published:** 2026-03-23

**Authors:** The Equi-Injury Group, Justine Davies, Leila Ghalichi

**Affiliations:** 1 Department of Applied Health Sciences, University of Birmingham, Birmingham, UK

**Keywords:** Injury

## Abstract

**Background:**

Injury burden is high in low-income and middle-income countries (LMICs). Delays in accessing definitive care after injury beyond the ‘golden’ hour or 2 hours worsen outcomes. We examined delays in accessing definitive healthcare after injury and whether their magnitude and associations differ across four diverse LMICs: Ghana, Pakistan, Rwanda and South Africa.

**Methods:**

Across 19 hospitals providing definitive care for injuries in urban or rural settings, we enrolled patients with moderate to severe injuries who were hospitalised for at least 12 hours. The time between injury and admission for definitive care and perceived reasons for delays in seeking and reaching care were captured. The association between more than 1-hour delay to reaching definitive care and age, sex, education, wealth, injury mechanism or severity, prior healthcare encounters, ambulance transport, the hospital type and catchment area was evaluated in a multivariable model. Patients’ perceived reasons for delay in seeking and reaching care were described. Findings were compared between countries.

**Result:**

Data on delays were available for 8331 patients, of whom 57.3% experienced delays exceeding 1 hour. Prior healthcare encounter before definitive care showed the strongest association with delay (OR: 8.44, 95% CI 7.41 to 9.60). Delays were associated with older age, less education and wealth, greater injury severity, urban (vs rural) catchment area, ambulance transport, injury mechanism due to falls or fire (vs road traffic collision) and tertiary (vs secondary) hospital admission in the adjusted model. Ghana and Rwanda showed the lowest and highest odds of delays compared with South Africa, respectively. Only 18.8% of patients perceived being delayed, most citing unawareness of urgency and ambulance unavailability as reasons.

**Conclusions:**

Most injured patients do not arrive at definitive care within the critical golden hour, with delays inequitably affecting the population. Improvements in pathways to care are needed to reduce delays across healthcare systems.

**WHAT IS ALREADY KNOWN ON THIS TOPIC**

- Delays in accessing care after injury are associated with increased patient morbidity and mortality. Although the burden of injury is high in low-income and middle-income countries (LMICs), there is a lack of multicountry evidence on the extent of both actual and perceived delays experienced by individuals with various types of moderate to severe injuries.

**WHAT THIS STUDY ADDS**

- Using data from more than 8000 patients admitted with diverse injury mechanisms and types in hospitals with urban and rural catchment areas, we show the high and inequitable prevalence of delays in arriving at definitive care after moderate to severe injuries across multiple LMICs. Despite the high prevalence of actual delays, few patients perceived any delays in seeking and reaching care. The key determinant of delays was healthcare encounters before reaching the hospital providing definitive care, while use of ambulances was associated with more delays than other modes of transport.

**HOW THIS STUDY MIGHT AFFECT RESEARCH, PRACTICE OR POLICY**

- Prevalence of delays in getting to a hospital providing definitive care after injuries is high and inequitably experienced. There are key factors including pathways and transport taken to get to care which affect delays. Most literature on LMICs focuses on care provided in healthcare facilities; however, delays in accessing healthcare facilities are key to address in order to reduce deaths and disability for those who are inevitably injured.

## Introduction

 Injuries account for 8% of global deaths, with populations in low-income and middle-income countries (LMICs) being disproportionately affected.^1 2^ It is estimated that one-third of mortality after injuries in LMICs is avoidable and 40% of these avoidable deaths are due to lack of timely access to quality care.^3 4^ The combination of high burden of injuries and avoidable deaths in LMICs calls for deeper understanding of health system provisions for injury care in these countries to develop effective policies and strategies.^5^

Timely access to care is a key measure of a health system’s provision of quality care.^6^ Delays to quality care are typically considered at the stages of seeking, reaching, receiving and remaining in care.^7 8^ Reducing delays at the stages of seeking and reaching definitive care, which we define as a care appropriate for severity of injury, is especially important in reducing adverse outcomes in time-sensitive conditions, of which injury predominates.^6 9 10^ It is also known that delays in seeking or reaching definitive care are associated with higher costs and financial burden, culminating in an increased risk of experiencing catastrophic health expenditure.^11^ Despite a wide spectrum of injury severity and mechanisms, evidence suggests that health outcomes are worse when definitive care is reached after the first ‘golden hour’ or the first 2 hours following injury.^12 13^ There are some controversies over associations between delays in getting to definitive care and patient outcomes in high-income countries.^12^ However, the concept of the golden hour or 2 hours for access to care after injury is sufficiently embedded into injury care policy and practice, and many LMICs are adopting these time thresholds as targets to demonstrate quality injury care, including investment in ambulance services to facilitate and expedite delivery of definitive care.^14^

Despite interest in reducing the time to get to definitive care in many LMICs, there is limited knowledge on the temporal delay, the stage at which the delay occurs (seeking, reaching or receiving care), and the individual, injury or health system factors associated with experienced delays.^15^ Much of the research done to date has focused on a single injury type.^16^ The stage of receiving care once the patient has arrived at the health facility, including the time between admission and start of time-sensitive procedures, has also received attention.^15^ But this neglects the fact that delays experienced during seeking and reaching care contribute substantially to overall delays and require different policies and strategies to overcome.^3 4 17 18^

To assist in developing commensurate and contextually relevant interventions to reduce delays to accessing definitive care after injury, our aim was to understand the magnitude and identify the risk factors of delays to access to definitive healthcare after injury, and whether these differ across four contextually diverse LMICs. To fulfil our aim, we describe the delays experienced by injured patients, their associated factors and perceived reasons for these delays in a sample of injured patients in four contextually different LMICs, Ghana, Pakistan, Rwanda and South Africa.

## Methods

This cross-sectional study is nested within the Equi-Injury cohort study, investigating equitable access to injury care in Ghana, Pakistan, Rwanda and South Africa. These four LMICs were selected due to their high injury burden and diverse economic, social and health system characteristics at the time of planning the study (online supplemental appendix 1). The full methods and selection criteria are described in a separate publication.^19^ In brief, data were collected from consecutive consenting injured patients admitted to secondary or tertiary referral facilities for at least 12 hours. The threshold of 12 hours was selected *a priori* on discussion between investigators as a proxy for the patient having suffered a moderate to severe injury, given that note-keeping in many of the included hospitals was likely to be incomplete and even if available, formal trauma severity scores, while reliable indicators of mortality, do not predict all dimensions of severity, like disability, time off work or loss of earnings.^20^ The study facilities were purposively selected to be as nationally representative as possible of hospitals that can provide definitive care for injured patients living in urban or rural areas, while being feasible for data collection.

Trained research assistants collected data prospectively between February 2023 and August 2024, using the REDCap electronic data platform^21^ by consulting patient medical records (electronic or paper-based) and asking direct questions of patients or their caregivers. There were 19 hospitals from which data were collected in total, 6 in Ghana, 5 in Pakistan, 4 in Rwanda and 4 in South Africa. At least two hospitals with a rural catchment area were included from each country. Data were cleaned and locked for analysis in November 2024.

### Variables

Our primary outcome was a delay in arriving at definitive healthcare of more than 1 hour after the injury occurrence. Our secondary outcome was a delay of more than 2 hours. Data to inform derivation of delays were captured as less than 1 hour, 1–2 hours, 2–5 hours, 5–12 hours, 12–24 hours, 1–2 days and longer than 2 days. Arriving after the first and second hour was defined as experiencing the primary and secondary outcomes.

Sociodemographic variables were age (in years), sex (male or female), education (categorised into no formal education, primary completed, secondary or higher completed), wealth (as quintiles based on household assets, derived using principal component analysis), and population predominately served by the hospital (rural or urban, assigned based on local expertise). Household assets used to derive wealth index included size of household, electricity source, type of toilet, main water source, household goods (smart phone, radio, television (TV), computer, refrigerator, dishwasher, clothes washing machine, tumble dryer), type of transport used by household members, roof material and external wall material.^19 22^

Injury variables were mechanism of injury (interpersonal violence (IPV), road traffic collisions (RTC), fall over or off something, hit by a falling object, fire or heat and other (including but not limited to chemical injury and inhalation or swallowing toxic materials)) and type of injury (as bone or joint, neurotrauma, cuts or soft tissue, other isolated injuries and polytrauma). We derived the Kampala Score, where data were available on age, systolic blood pressure, respiratory rate, neurological status and number of serious injuries, to further stratify injuries by severity (categorised as mild, moderate or severe). However, note that patients had already been recruited to the study based on our—project team—definition of moderate to severe injury warranting formal admission to hospital for longer than 12 hours.^23^ Health service variables were definitive care hospital level (secondary or tertiary), mode of transport as ambulance or other (other includes privately owned or hired motor vehicle, public transport (bus/local equivalent), bicycle, police car, walking or carried (eg, on stretcher)) and the number of facilities or healthcare encounters (hereafter, prior healthcare encounters) attended prior to arriving at the definitive facility (none, 1, 2 or more). Prior healthcare encounters included primary care, district hospital, teaching or regional hospital, or pharmacies, bone setters or traditional healers.

Patients were asked whether they perceived that there had been a delay in seeking care and their perceived reasons if so (unaware of need to get to care quickly, fear of safety to travel to hospital, necessity to ask for permission from relatives or community, fear of costs of transport to hospital being unaffordable, fear of costs of care being unaffordable, lack of trust that the care received would be good, preferring bone setter/traditional healer, other duties that need to attend to, fear of losing job or free text for other reasons). Patients were also asked whether they perceived any delay in reaching care and their perceived reasons if so (no money to pay for transport or care, lack of ambulances, lack of other transport, transport being too slow, poor road infrastructure or free text for other reasons).

### Statistical analyses

This was a secondary analysis of an existing dataset, and thus, a power calculation was not done. The power calculation for the full study is documented in the protocol.^19^ Characteristics of participants are summarised across all countries using descriptive statistics as appropriate. We graphically show reported delays, the number of prior healthcare encounters before arriving at definitive care, perceptions of whether there was any delay by reported time to get to the definitive facility, and the reasons for perceived delays. A χ² test was used to evaluate the association between categorical variables, including reported and perceived delays, and ambulance and injury severity. We report the use of ambulances by injury severity and country.

We used multivariable logistic regression to examine associations with reported delay as no delay (arriving within an hour after injury) versus delay (arriving after the first hour). Covariables identified a priori were age, sex, education, wealth, hospital catchment area, injury mechanism, prior healthcare encounter (vs arriving directly at the definitive care), arriving by ambulance and definitive hospital type. Country was included in the model as a fixed effect. We did not include type of injury in the model, given that the severity rather than the type should determine the rapidity of arrival to definitive care. We evaluated multicollinearity in the model using variance inflation factor and checked two by two correlations. The model was fitted to data from each country separately. We repeated the multivariable logistic regression using our secondary outcome variable of delay of more than 2 hours versus no delay (arrival at definitive care before 2 hours).

We did seven sensitivity analyses. First, we repeated the main analysis for the primary and secondary outcomes using modified Poisson regression, in case the outcomes were not rare. Second, we repeated the main analysis using multiple imputation by chained equations, using the mi package in Stata V.19. The number of imputed datasets created was equal to the percentage of participants with at least one variable missing. The primary outcome was not imputed. Third, to understand reasons behind findings after multiple imputation, we repeated this model with an interaction term between country and arriving by ambulance. Fourth, we repeated the complete case (main) analysis with the interaction term between country and arriving by ambulance. Fifth, we repeated the main analysis after removing the hospital catchment area, given that this might not accurately represent the habitation of individual patients. Sixth, we added injury type to the main analysis to understand if this did influence delays. Seventh, to understand the effect of taking an ambulance directly to definitive care from the scene versus interfacility transfer, we both repeated the main analysis in those who arrived directly to definitive care and included an interaction term between prior healthcare encounter and arriving in an ambulance in the model. Sensitivity analyses two to seven were done using the primary outcome.

## Results

Of 9720 patients who were eligible to be recruited in the main study, 312 did not consent to take part, 550 left and 299 died before data collection. Therefore, 8559 participants were eligible for this analysis, of whom data on delay to care following injury were available from 8331 patients (97.3%). Of participants with data on delays to care, 1947 (23.4%) were female, and the median age was 30 (IQR: 20–43) years. Primary education was completed by 3059 (36.8%) and 5208 (62.5%) were admitted to hospitals serving predominantly urban catchment areas. RTC accounted for 4156 (49.9%) of the injuries, followed by falls and IPV. In 6690 patients with Kampala Trauma Score calculated, 2860 (42.8%) were categorised as moderate or severe, the others were re-categorised as mild (table 1, online supplemental appendix 2).

**Table 1:** Participant characteristics in injured patients in four low-income to middle-income countries

Variable	N (%)
Sex (n=8300)	
Female	1947 (23.4%)
Male	6363 (76.6%)
Age median, (IQR) (n=7979)	30 (20, 43)
0–10	928 (11.4%)
11–20	1179 (14.4%)
21–30	1987 (24.3%)
31–40	1767 (21.7%)
41–50	1015 (12.4%)
51–60	635 (7.8%)
61–70	382 (4.7%)
71–80	182 (2.2%)
>80	86 (1.1%)
Education (n=8319)	
No formal education	2516 (30.2%)
Primary completed	3059 (36.8%)
Secondary completed or more	2744 (33.0%)
Wealth (n=7268)	
First quintile	1467 (20.2%)
Second quintile	1442 (19.8%)
Third quintile	1457 (20.0%)
Fourth quintile	1453 (20.00%)
Fifth quintile	1449 (19.9%)
Hospital catchment area (n=8331)	
Rural	3123 (37.5%)
Urban	5208 (62.5%)
Mechanism of injury (n=8325)	
Interpersonal violence	1390 (16.7%)
Road traffic accident	4156 (49.9%)
Fall	1736 (20.9%)
Hit	284 (3.4%)
Fire or heat	248 (3.0%)
Other	511 (6.1%)
Injury severity (n=6690)	
Mild	3830 (57.2%)
Moderate	2555 (38.2%)
Severe	305 (4.6%)
Hospital type (n=8331)	
Secondary	1606 (19.3%)
Tertiary	6725 (80.7%)
Type of injury (n=8275)	
Orthopaedic injury	3364 (40.7%)
Neurotrauma	1320 (16.0%)
Cuts and soft tissue	1135 (13.7%)
Other isolated injuries	778 (9.4%)
Polytrauma	1678 (20.3%)
Prior healthcare encounters (n=8326)	
No	3819 (45.9%)
Yes (total)	4507 (54.1%)
1 facility	3793 (45.6%)
2 facilities or more	701 (8.6%)
Type of prior healthcare encounters*	
Primary care	1762 (21.1%)
District hospital	2745 (32.9%)
Teaching or regional hospital	464 (5.6%)
Pharmacy	4 (0.05%)
Bone setter or traditional healer	21 (0.3%)
Mode of transport (n=8331)	
Ambulance	3823 (45.9%)
Ambulance (direct to definitive facility)	1217 (14.6%)
Ambulance (interfacility transfer)	2606 (31.3%)
Other mode of transport	4508 (54.1%)
Arrival time (from injury) (n=8331)	
During first hour	3558 (42.7%)
After first hour	4773 (57.3%)
During second hour	1960 (23.5%)
After second hour	2813 (33.8%)
Perceived delay (n=8299)	
No	6740 (81.2%)
Yes	1559 (18.8%)
Country (n=8331)	
Ghana	2129 (25.6%)
Pakistan	2552 (30.6%)
Rwanda	1884 (22.6%)
South Africa	1767 (21.2%)

Table 1 shows characteristics of the 8331 patients with data on delays in the Equi-Injury study. The denominator for individual variables is shown in parentheses after the variable name.

* Not mutually exclusive (ie, patients could have had multiple healthcare encounters).

† Based on Kampala Trauma Score.

**Figure 1:**
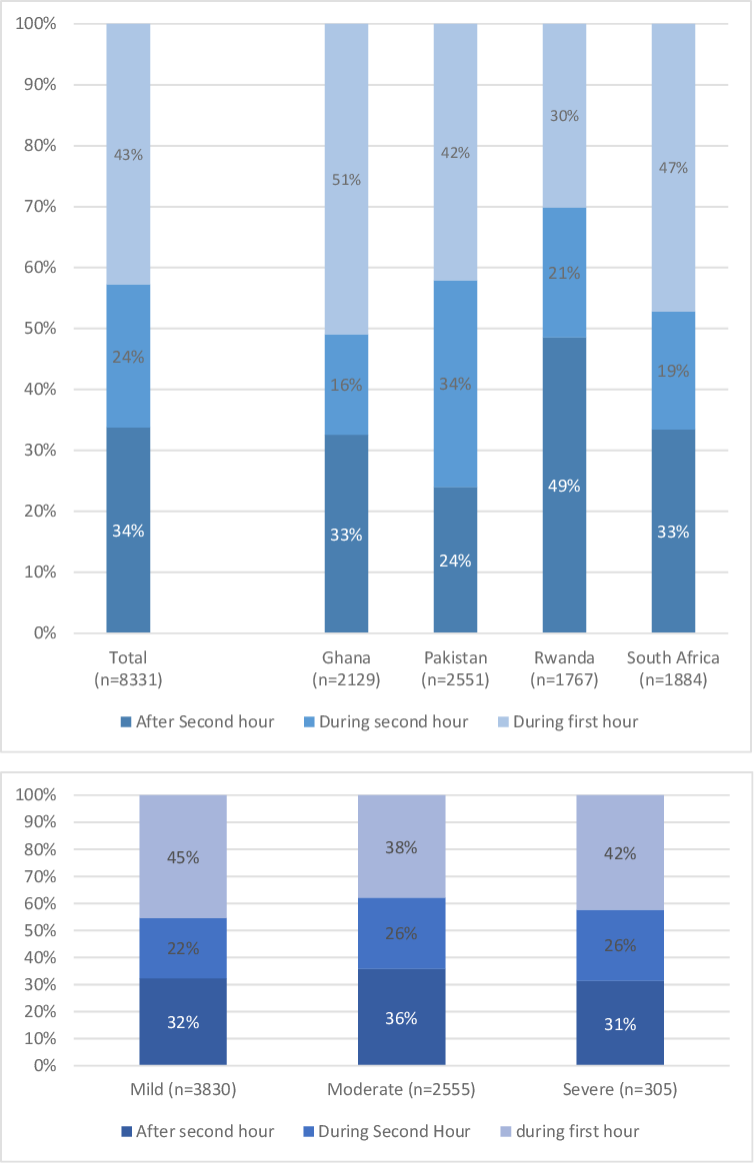
Arrival time at the definitive facility, shown as arrival during the first or second hour, or after the second hour after injury, (A) in 8331 total sample and by country and (B) based on injury severity in 6690 patients.

Of 8331 patients who had data available, a delay of more than 1 hour was reported by 4773 (57.3%) patients, and 2813 (33.8%) patients reported a delay of more than 2 hours (figure 1). In total, 3823 (45.9%) patients were transported by ambulance; 1217 (14.6%) were directly transported to the definitive care facility by ambulance, whereas 2606 (31.3%) were transferred by ambulance from another facility (table 1). Among 6690 patients with Kampala Trauma Score, 45.4%, 37.9% and 42.3% of patients with mild, moderate and severe injuries arrived during the first hour after injuries (figure 1). Increased injury severity was associated with use of ambulance transport; in 6690 patients, ambulances were used for 42.0%, 50.5% and 67.2% for mild, moderate and severe cases, respectively (p<0.001). Ambulances were used for 20.5% of 2129 injured persons in Ghana, 51.2% of 2551 in Pakistan, 49.0% of 1767 in South Africa and 64.4% of 1884 in Rwanda.

**Figure 2:**
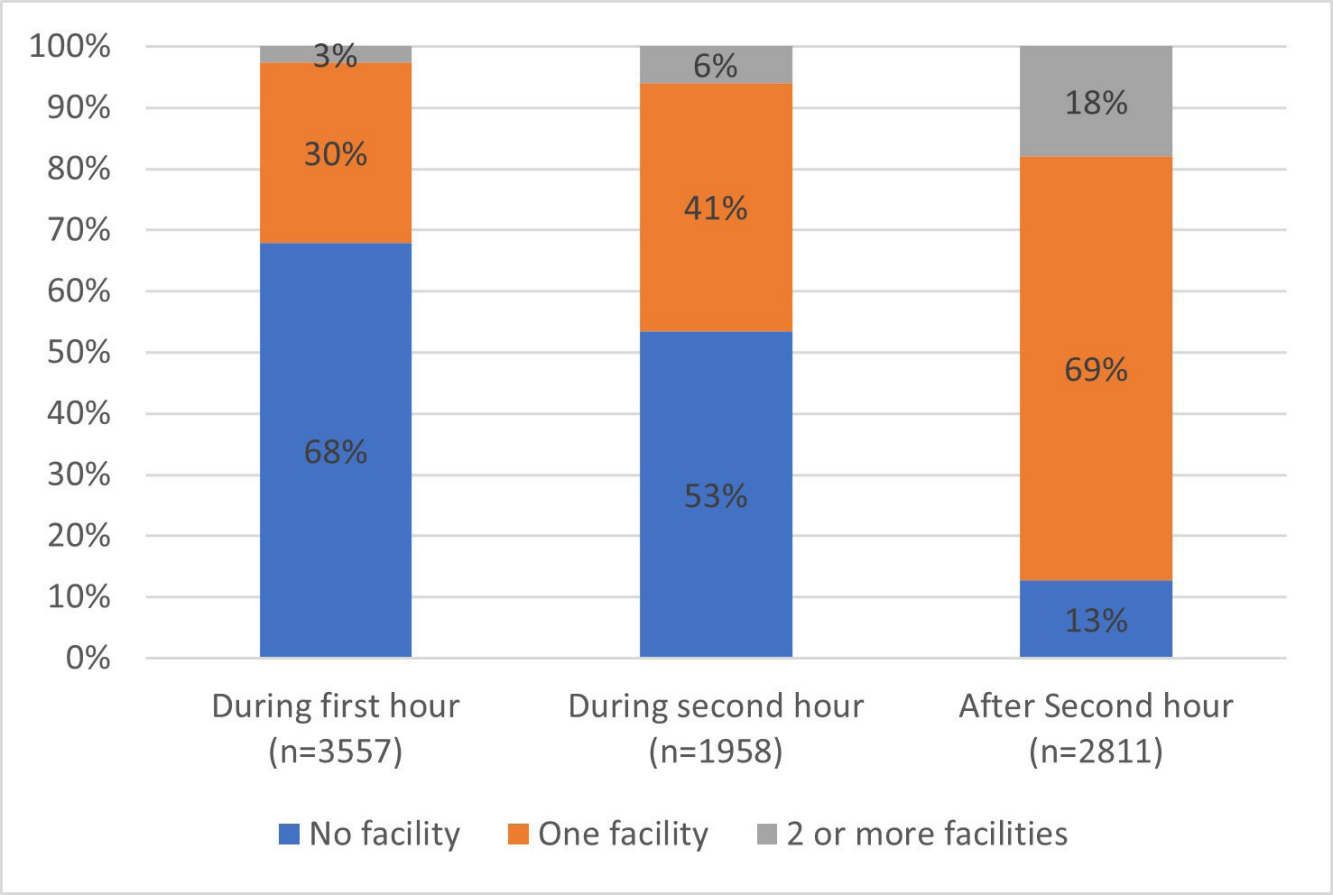
Number of prior healthcare encounters by time of arrival at the definitive facility, shown as arrival during the first or second hour, or after the second hour after injury.

In 8326 participants with data on prior healthcare encounters, 3819 (45.9%) arrived directly at a definitive care facility. Prior healthcare encounters were mainly at district hospital (2745, 32.9%), teaching or regional hospitals (464, 5.6%) or primary care (1762, 21.1%). Only 21 patients (0.3%) reported visiting a bonesetter or traditional healer and 4 (0.05%) patients reported visiting a pharmacy (table 1). The type of healthcare encounters visited prior to arriving at definitive care varied by country (online supplemental appendix 3). While 67.9% of the 3557 and 53.5% of the 1958 arriving during the first or second hour after injury had had no prior healthcare encounter, respectively, only 12.7% of those arriving after the second hour had not had a prior healthcare encounter (figure 2).

**Figure 3:**
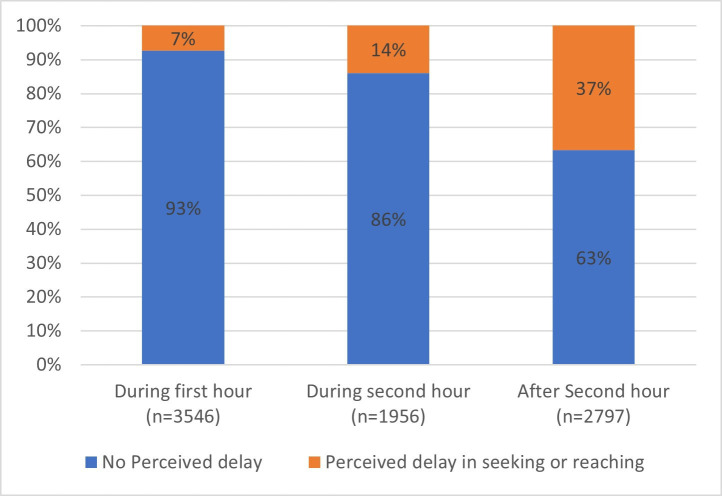
Perceived delay in seeking or reaching care by time of arrival at the definitive facility, shown as arrival during the first or second hour, or after the second hour after injury.

Among the 8299 participants with data on perceived delay, 1559 (18.8%) reported some delay in seeking or reaching care or both. The number of patients who reported a delay between the injury and arrival at the definitive facility was larger than those who perceived having a delay; among those reporting that they arrived in the first and second hour after injury, 7.4% and 13.9% perceived any delay, respectively. For those who had more than 2 hours delay, 36.9% perceived no delay (p<0.001) (figure 3).

The reasons for perceived delays are shown in online supplemental appendix 4. Of the 8304 participants who answered the question on perceived delay in seeking care, 1151 (13.9%) perceived some delay in seeking care. The most common perceived reason was being unaware of the need to get care quickly. This was reported by 685 (59.5% of 1151), varying from 41.4% with IPV to 70.6% in fire or heat as the mechanism (online supplemental appendix 5). Considering different injury types, the largest proportion reporting this as a reason was in orthopaedic injury (64.1) and neurotrauma (63.4%). Of 8326 participants with data on perceived delay in reaching care, 1042 (12.5%) reported some delay in reaching care. The most common perceived reason for a delay in reaching care was lack of ambulances (56.2% of the reported reasons) (online supplemental appendix 4).

In the multivariable analysis, including 5808 patients with complete data, the highest odds of experiencing a delay of more than 1 hour before arrival at the definitive facility was associated with prior healthcare encounters versus coming direct to the definitive facility (adjusted OR (AOR) 8.44, 95% CI 7.41 to 9.60) (table 2). Older age, lower levels of education, greater severity of injury, lower wealth, urban catchment area, arrival by ambulance and being admitted to a tertiary hospital were all associated with greater odds of delay than their comparator (table 2). Country was also significantly associated with delay, with the highest odds of delay in patients from Rwanda compared with South Africa (AOR 3.05; 95% CI 2.4 to 3.87). Higher odds of delay were found in the injury mechanisms of fall and fire, compared with RTC.

**Table 2:** Multivariable analysis showing association of experiencing delays to definitive care after moderate to severe injury

	1-hour delay			2-hour delay		
	AOR	95% CI	P value	AOR	95% CI	P value
Age	1.01	1 to 1.01	<0.001	1.01	1.01 to 1.02	<0.001
Sex (Ref: female)	0.87	0.75 to 1.02	0.092	0.95	0.8 to 1.11	0.504
Education (Ref: secondary or higher)						
No formal education	1.02	0.86 to 1.21	0.779	1.29	1.08 to 1.53	0.005
Primary completed	1.28	1.1 to 1.48	0.001	1.21	1.04 to 1.42	0.015
Country (Ref: South Africa)						
Ghana	1.61	1.3 to 2	<0.001	1.76	1.41 to 2.2	<0.001
Pakistan	0.87	0.71 to 1.06	0.16	1.13	0.92 to 1.4	0.25
Rwanda	3.05	2.4 to 3.87	<0.001	2.78	2.21 to 3.49	<0.001
Wealth quintiles (Ref: Q5)						
Q1	1.53	1.24 to 1.9	<0.001	1.39	1.12 to 1.74	0.003
Q2	1.56	1.27 to 1.9	<0.001	1.34	1.09 to 1.65	0.005
Q3	1.44	1.18 to 1.75	<0.001	1.25	1.02 to 1.54	0.033
Q4	1.02	0.85 to 1.24	0.803	1.03	0.84 to 1.27	0.761
Mechanism (Ref: RTC)						
Interpersonal violence	1.08	0.89 to 1.32	0.431	1.19	0.97 to 1.46	0.087
Fall	1.39	1.16 to 1.66	<0.001	1.41	1.18 to 1.7	<0.001
Hit by a falling object	1.34	0.92 to 1.95	0.133	1.84	1.28 to 2.66	0.001
Fire or heat	1.72	1.04 to 2.86	0.036	1.54	0.95 to 2.48	0.079
Other	1.45	1.09 to 1.94	0.012	1.33	1.01 to 1.77	0.045
Injury severity (Ref: mild)						
Moderate	1.28	1.11 to 1.47	<0.001	1.01	0.88 to 1.17	0.883
Severe	1.37	1.02 to 1.84	0.038	0.72	0.52 to 0.98	0.036
Prior healthcare encounter (Ref: no prior health encounter)	8.44	7.41 to 9.60	<0.001	12.47	10.61 to 14.66	<0.001
Transport by ambulance (Ref: other modes of transport)	1.41	1.22 to 1.62	<0.001	1.21	1.05 to 1.4	0.008
Urban catchment area (Ref: rural)	1.43	1.17 to 1.75	0.002	1.43	1.15 to 1.78	0.001
Tertiary hospital (Ref: secondary)	1.34	1.06 to 1.68	0.015	1.18	0.92 to 1.52	0.199

Table 2 shows results of the multivariable analysis with the primary outcome (more than 1-hour delay) and secondary outcome (more than 2-hour delay). 5808 complete cases were included.

AOR, adjusted OR; RTC, road traffic collisions.

Some deviations from the cross-country model were observed in country-specific models (online supplemental appendix 6). In Ghana, injury severity and hospital type were not associated with delays. In Pakistan, being female was associated with greater odds of delay; however, being transported by ambulance or having no formal education was associated with lower odds of delay, and hospital type and injury mechanism was not associated with delays. In Rwanda, age, education, wealth, injury mechanism and severity were not significantly associated with delays. In South Africa, wealth and injury severity were not significantly associated with delays.

Considering the secondary outcome of a 2-hour delay to arrival at the definitive facility, the odds remained similar to those seen when using 1-hour delay, except for injury severity, where greater severity of injuries was protective against 2-hour delays (AOR 0.72, 95% CI 0.52 to 0.98). Additionally, the mechanism of fire was replaced by being hit as that with the greatest odds of delays compared with RTCs and being admitted to a tertiary hospital versus a secondary hospital was no longer significant in the model (table 2).

The outcome was not rare, and the findings from the first sensitivity analysis using a modified Poisson approach are presented in online supplemental appendix 7. The pattern and statistical significance of associations remained similar; however, the association between tertiary hospital admission and delay was attenuated and no longer statistically significant. Running the multivariable model for the primary outcome on the imputed dataset in the second sensitivity analysis yielded similar directionality and significance for most variables compared with the main—complete case—analysis, but the association between ambulance transport and delays was no longer observed (AOR 0.9, 95% CI 0.81 to 1.01) (online supplemental appendix 8). In the third sensitivity analysis to explore the reason for the change in the significance of taking an ambulance when using multiple imputation, adding the interaction term between country and ambulance transport showed that in Pakistan, ambulances were associated with a reduction in delays, whereas the association between ambulances and delays was no longer significant in Rwanda (online supplemental appendix 8). This finding was also confirmed in the fourth sensitivity analysis, when running the main model using complete cases and including the interaction term (online supplemental appendix 8). In the fifth sensitivity analysis, after removing the catchment area from the main model, the directionality and strength of the relationships did not change (online supplemental appendix 9). Including injury type in the model, in the sixth sensitivity analysis, resulted in the association between severity and a delay being non-significant; the directionality and significance of other associations was similar to the main model (online supplemental appendix 9). When repeating the analysis in the subgroup who went directly to the definitive care facility (with no prior healthcare encounter) in the seventh sensitivity analysis, transport by ambulances continued to show a substantial and significant association with delays compared with other modes of transport. The directionality of other relationships mainly remained similar to those seen in the main model; notable differences were that Ghana and Pakistan now showed significantly lower odds of delays than South Africa (online supplemental appendix 10). The inclusion of an interaction term between having a prior healthcare encounter and arriving by ambulance in the main model showed a significant protective effect (AOR: 0.61, 95% CI 0.47 to 0.79), indicating that although the ambulance remains a determinant of delays, the association of ambulance use and delays is significantly weaker in those with prior healthcare encounters (online supplemental appendix 10).

## Discussion

Across all four countries, we show a high frequency of delays in arriving to the definitive care facility after injuries. Most patients did not arrive at definitive care within the golden hour, and a large proportion were delayed for more than 2 hours. Patients from Ghana had the lowest prevalence of delays, while Rwanda experienced the greatest. Despite a high proportion of people arriving after the golden hour, most patients did not perceive that they had experienced a delay in getting to definitive care. This was even the case for patients who arrived after the second hour after injury. Among those who did perceive being delayed, being unaware of the need to get to care or lack of ambulances were the most frequently given reasons for the delay in seeking and reaching care, respectively. When considering all variables associated with a delay together, having prior healthcare encounters before arriving at definitive care was associated with the greatest odds of a delay. Other factors were increasing age, lower wealth or education, taking ambulance transport, going to a hospital with an urban catchment area and injury mechanisms of fall or fire. Increasing injury severity was associated with increased odds of delay in getting to the definitive hospital after the first hour.

Both 1-hour and 2-hour delays were frequent in the injured patients in our study. However, the odds of a delay of more than 2 hours were lower in patients suffering severe injuries, compared with mild injuries, while the odds of a delay of more than 1 hour were higher in those with severe injuries than those with mild injuries. While the ‘golden hour’ for getting patients to care after injury is a long-standing benchmark in the health-system literature and commonly applied in high-income settings,^9^ the Lancet Commission on Global Surgery (LCoGS)—which focused on care in LMICs—proposed a 2-hour time frame for getting patients who had severe injuries to care to minimise complications and mortality.^13^ The literature suggests that outcomes for patients are improved if patients get to facilities capable of providing definitive care within the first hour after injury; however, this is not always the case.^24^ Additionally, the feasibility of achieving this target in LMICs is questionable—which is one of the reasons for the threshold set by LCoGS being 2 hours. Nevertheless, that we showed that patients with more severe injuries were more likely to reach definitive care within 2 hours is encouraging that these targets might be feasible.

Over half of the patients had prior healthcare encounters before arriving at the definitive facility. Although this variable included visiting a traditional medical practitioner, bonesetter or a pharmacy, the numbers of patients who had visited these providers was very small. Our findings add to a small but growing body of literature suggesting similar issues of going to lower levels of healthcare facility prior to arrival at definitive care are experienced elsewhere.^16 25^ This might reflect injury referral guidelines requiring stabilisation or assessment of the injured at the nearest facility, or potentially, a lack of patient or community knowledge about which facilities are most appropriate for treatment of moderate to severe injuries. Ideally, patients should go directly to the facility that has the capability to treat them. This can be achieved by the development of policies and referral pathways to triage patients to appropriate facilities, as seen, for example, in the use of trauma centres which directly receive patients who have severe injuries in the UK.^26^ An alternative is to improve the readiness of lower-level facilities to deal with moderate to severely injured patients. Neither of these solutions is straightforward to implement. Public health information campaigns could encourage patients to go directly to a definitive treatment facility. However, this could risk overwhelming secondary and tertiary facilities with mildly injured persons who could be treated at lower-level facilities.^27^ On the other hand, improving the readiness of lower-level facilities to deal with moderate to severely injured patients would require substantial investment and may be inefficient.^28^ Interestingly, in our study, most patients who had a prior healthcare encounter had visited district or tertiary hospital, and it is likely that they were referred to the definitive care facility due to need for specialised services, or a lack of capacity at the initial facility. Further investigation into the reasons for referral within each country’s specific context could help clarify patient trajectories and distinguish between systemic barriers and expected pathways to definitive care. Only around 20% of patients in our study sought care first at primary care, suggesting that there was some patient or community recognition of the need for higher level of care, commensurate with the severity of the injury. Interestingly, the proportion of patients first attending primary care for their injuries varied by country, with a low proportion in Pakistan and the greatest proportion in Rwanda. The establishment of a community-based health insurance scheme, known as Mutuelle de Santé, by the Government of Rwanda, may reflect the necessity of some patients to first attend at primary care, as part of the requirements of this scheme.^29^,^33^ Other investigators have found higher initial primary care attendance rates in other countries, suggesting that pathways to care for injured persons vary depending on country context.^3 25^

Across study countries combined, the use of ambulances was associated with greater odds of delay compared with other forms of transport, even in patients transported directly from the scene to the definitive facility. However, when using multiple imputation and including an interaction term between country and ambulance transport, it was clear that this association is different across countries, with ambulances versus other transport being associated with delays in Ghana and South Africa, there being no association in Rwanda, and ambulances being associated with lower odds of delay in Pakistan. Results from individual countries are similar to that of the sensitivity analysis, apart from Rwanda still showing significant odds of delays when ambulances are taken. There are several potential reasons for findings that ambulances may be associated with a delay. Ambulances may be called for cases which are more severe, and the delays caused by dealing with severity at the scene; however, controlling for severity using the Kampala score did not impact on this association. Additionally, ambulance response time (ie, time it takes for an ambulance to arrive at the scene after the emergency call) is generally high in many low-resource settings with ambulances often stationed to optimise coverage and not response times.^34^ Hence, unless the ambulance response time is negligible, taking alternative available transport might be quicker. Once the patient is en route to hospital, the speed at which the ambulance can travel is often no different to that of other traffic, given that ambulances might not have right-of-way on roads. This is a particular issue in countries with high levels of congestion on the roads.^35^ Additionally, ambulance dispatch services (which take calls and prioritise ambulances for dispatch) are often nascent in many LMICs, for example with minimally trained call takers and ambulance dispatchers, or the use of services, for example caller and ambulance dispatch triage tools, which are not developed for the contexts in low resourced settings.^36^ These issues are compounded by multiple possible emergency call numbers and fragmented ambulance providers, leading to delays in dispatching ambulances and triaging the patients to commensurate facilities.^37^

We interestingly saw divergent results between the study sites in Rwanda and Pakistan, both of which have established ambulance services which are either free of charge or covered by universal health coverage. Whereas in Rwanda, ambulances were associated with a greater, or at least not better, odds of delays, in Pakistan, ambulances were consistently associated with a significant improvement in delays. There has been a large investment in government-funded emergency medical services in Sindh province (Pakistan), where the study took place, including a large ambulance dispatch centre, high standard of personnel training, a special emphasis on placing ambulances closer to areas with high risk of injuries and development of care pathways encouraging direct transport from scene to tertiary facilities.^38^ Additionally, an ambulance Right of Way campaign was started in 2016 with the help of several TV and radio channels.^39^ These initiatives might have contributed to the lower odds of delay when using ambulance transport in Pakistan. Although ambulance services exist in Rwanda, at the time of the study, these were focused in Kigali, there were few ambulances, the dispatch system was nascent, and training for ambulance and dispatch personnel was not consistent.^35^ Even in high-income countries, with mature ambulance services, informal transport may be associated with fewer delays in accessing care than taking ambulances.^40^,^42^ Hence, before investing in ambulance services, countries need to consider the rationale for investment and the full health system ecosystem in which the services will sit. Investing in quality and efficient prehospital emergency care systems is resource intensive.^43^ It requires investment in trained human resources, medical equipment and technologies, data infrastructure, an organised ambulance dispatch centre, an ambulance fleet, and policies to ensure that ambulances have the right of way on roads. Systems from HICs are not necessarily transferable to LMIC settings, given lower resources and different health system contexts.

We found that ambulances were most frequently used for interfacility transfers, rather than primary transport of patients from the scene of the injury or home, with nearly two-thirds of injured patients arriving via ambulance having previously visited another facility. This fits with knowledge from our country partners. For example, in Ghana, more than 80% of the capacity of national ambulance service is used for inter-facility transfers,^44^ and despite the expansion of national ambulance service and significant strides in accessibility across the entire country in recent years,^44 45^ ambulances were rarely used for patient transport from the scene to hospitals in our study.

Patients in our study also experienced higher odds of delays if the definitive facility was a tertiary hospital compared with a secondary hospital. While the effect of prior facility attendance could partially explain this, the effect was seen even when controlling for prior facility attendance. This finding may be reflective of the greater distance from the site of injury to a tertiary referral hospital, but unfortunately, distance from injury to hospital was not collected in our study. Our finding might also reflect that tertiary hospitals are most likely to be in urban areas, aligning with the finding that urban catchment, potentially related to traffic volume, was also associated with longer delays. Some, although not all, evidence from other LMICs suggests that direct transfer of patients to tertiary facilities following injury is associated with survival benefits, and the survival benefit is one of the reasons behind the focus on treating severely injured patients in major trauma centres in high income countries.^46^ While the distance to travel to major trauma centres is often greater than going to a local facility, the concentration of expertise and equipment is thought to outweigh any increase in travel time.^47^ In this analysis, we cannot show if the delay associated with being treated in a tertiary facility is associated with patient clinical outcomes. However, considering the literature in totality suggests caution should be applied in LMICs aiming to reduce their prehospital referral times without considering the capability of the hospital to which the patient is transported.

Injury mechanism was another determinant of delays. RTCs were generally associated with lower odds of delay than other mechanisms. This might be due to their occurrence in public locations which facilitate prompt transport, although lack of legal and moral obligations of bystanders and fear of reprisal of good Samaritans will often adversely impact on time to reach care even in the most well-connected RTC site.^48^ Falls and fire, on the other hand, are associated with greater odds of delays. Studies have shown that these injuries happen more frequently at homes and hence may be at a distance from available transport.^49^ The concept of the ‘long lie’, when individuals are unable to stand up on their own after falls, is also becoming established.^50^

We found substantial and significant differences when considering whether delays were experienced equally among patients. Economic status was consistently associated with delays, whether considered using education or wealth as an indicator. This could be due to several factors, for example, people with lower economic means might live in areas further from facilities providing affordable care. They might also have limited ability to pay for transport or care, delaying the decision to seek care.^51 52^ Indeed, we found that fear of costs of transport or care being unaffordable and lack of financial resources to pay for transport were two reasons expressed by respondents for delays in seeking and reaching care. We also found (data not shown) that people in the higher wealth quintiles visited fewer prior facilities than those who were poorer. Those with greater education may have more knowledge of the need to get to definitive care quickly, which potentially aligns with our finding that the most common reason for a perceived delay in seeking care was unawareness of the need for urgent care. Interestingly, and in contrast to other studies in similar settings, visiting bonesetters or traditional healers prior to seeking formal healthcare as the perceived reason for delay was uncommon in our study.^53^ This might reflect knowledge among patients of the injury’s severity, given that our sampling strategy was to recruit hospitalised patients with moderate to severe injuries. In the whole sample and in most of the study countries, sex was not associated with delays. However, in Pakistan, being female was associated with an almost 30% increase in chance of delay compared with males. Gender inequality in access to health in Pakistan has been well documented previously, reflecting existing cultural practices and lack of women’s agency that restrict women’s mobility and autonomy.^54^ Increasing age was associated with a small but significant increase in odds of a delay. This has been observed in various time-sensitive situations, with heterogeneous pathways in younger and older adults in traumatic brain injuries, strokes and myocardial infarctions.^55^,^58^

### Limitations

Our study includes patients hospitalised for at least 12 hours after admission as an indication of an injury being moderate to severe; thus, it does not include those who died before this timeframe or those discharged earlier, and the findings should be generalised accordingly. Additionally, although the study project leads agreed that being admitted to hospital for 12 hours was a good indicator of an injury being moderate or severe in the contexts (ie, it was bad enough to require management warranting a stay in hospital), in patients in whom Kampala score was calculated, the severity of the injury was often categorised as being mild. While simple and easy to use, hence suitable for low-resourced contexts, and associated with mortality, the Kampala score is criticised by some for having low agreement with other methods of judging injury severity. Given the ongoing discussions around the best trauma severity score to use to predict outcomes, we present the Kampala score to illustrate the spectrum of injury severities seen in our study, rather than to present an absolute severity.^20 59 60^ Patients reported the time gaps, and they were not objectively verified. However, given injury is a stochastic event and many people in LMICs do not use ambulance transport, capturing the actual time from the injury occurring to arrival at definitive care is highly challenging. We have not differentiated the duration of delay in seeking and reaching care, although we could shed light on the perceived reasons for these delays. The study does not include injury location and patient residence, both of which could further clarify the patient trajectory. We used the wealth index as a marker of socioeconomic status. However, this can be more indicative of wealth accumulation than readily available funds which could be used to pay for care. Income or expenditure might better reflect availability of funding for care; however, the wealth index is more reliably collected than either of these variables, given individuals in hospital may not be the main earners or spenders, and economies in many LMICs are often informal, with income and spending being highly variable across people and time. ^61^Additionally, in many LMICs, household goods are sold or lent out to pay for healthcare.^62^

## Conclusions

While most of the research and policy in LMICs is focused on delay in receiving care once the patient has reached the healthcare facility, previous evidence on the magnitude and determinants of delay in seeking and reaching care is limited. We report that delays in arriving at definitive care facilities are common among injured patients in LMICs. They result from both delay in seeking and reaching care and are often inequitable. The need to visit multiple facilities is an important source of the delays. Our finding that ambulances are not consistently associated with reduced delays and often associated with increased delays should act as a caution to healthcare planners considering investing in ambulance services for this purpose. Policies that strengthen the entire prehospital care system, and not just individual components such as ambulance services, along with more streamlined referral pathways and public health education on timely care-seeking, could help improve patients’ trajectories from injury to hospital admission and result in more effective injury care outcomes.

## Supplementary material

10.1136/bmjgh-2025-021659 online supplemental file 1

## Data Availability

Data are available on reasonable request.
