# Supplementary material for: Delays in seeking and reaching care for injured patients in four low-income and middle-income countries: a cohort study
Source: BMJ Glob Health. 2026 Mar 23;11(3):e021659. doi: 10.1136/bmjgh-2025-021659 (PMC13034212; doi:10.1136/bmjgh-2025-021659)
Supplement: online supplemental file 1 [file bmjgh-11-3-s001.docx]

**Appendix 1**. Partner countries’ economic and health expenditure profile at the time of study.

|  | **South Africa** | **Ghana** | **Pakistan** | **Rwanda** |
| --- | --- | --- | --- | --- |
| **Income Status** | Upper middle | Lower middle | Lower middle | Low |
| **HDI rank** (1) | 110 | 145 | 164 | 161 |
| **Gini index** (2) | 63 | 43.5 | 29.6 | 43.7 |
| **% GDP on health** | 8.3 | 4.2 | 2.9 | 7.3 |
| **External HE (% current)** | 1.4 | 8.9 | 10.3 | 35.9 |
| **Private HE (% current)** | 38.2 | 37.1 | 60.7 | 23.3 |
| **OOP HE (% current)** | 5.5 | 27.2 | 57.5 | 10.1 |

Table1. Information is based on world bank data at the time of study (https://data.worldbank.org/).

HDI: Human Development Index, GDP: Gross Domestic Product, HE: Health Expenditure, OOP: Out Of Pocket

At the time of study, the four countries had diverse development, geographical, health systems, cultural, and injury contexts.

- Ghana, with an estimated 8% of deaths and 7% of DALYs due to trauma, had a high rate of out-of-pocket (OOP) health expenditure.
- In Pakistan, injuries were estimated to cause 7% of deaths, with high OOP expenditure and no Universal health Coverage (UHC) in place, although the government had committed to UHC and an essential package of health services has been approved.
- In Rwanda, 10% of DALYs and 9% of all deaths were due to trauma and the government had introduced a health insurance scheme to cover services for the entire population, with contributions that are means-assessed.
- South Africa had a very high rate of trauma rate, estimated to cause to 13% of deaths, mainly from interpersonal violence with a high proportion of the population being uninsured and dependent on the public health system.

Appendix 2. Data availability for each of the variables in the study.

| Variable | N |  | % |
| --- | --- | --- | --- |
| Sex | 8300 |  | 99.6% |
| Age | 7979 |  | 95.8% |
| Education | 8319 |  | 99.9% |
| Wealth | 7268 |  | 87.2% |
| Hospital catchment area | 8331 |  | 100.0% |
| Mechanism of Injury | 8325 |  | 99.9% |
| Injury Severity | 6690 |  | 80.3% |
| Hospital type | 8331 |  | 100.0% |
| Type of Injury | 8275 |  | 99.3% |
| Prior healthcare encounters | 8326 |  | 99.9% |
| Mode of transport | 8331 |  | 100.0% |
| Arrival time (from injury) | 8331 |  | 100.0% |
| Perceived Delay | 8299 |  | 99.6% |
| Country | 8331 |  | 100.0% |

Appendix 3. The type of healthcare encounters prior to arriving at definitive care by injured people in the four countries

Appendix 2 shows the type of healthcare facilities visited by injured patients prior to arriving at the definitive care facility. Data were available for 4502 of 4507 patients who were eligible to answer this question, although some patients might have visited more than on facility prior to arriving at the definitive care facility.

Appendix 4. Perceived reasons for delay in seeking care (A) and reaching care (B) (patients could chose more than one reason for each)

|  |  |
| --- | --- |

Appendix 4 shows perceived delays in A. seeking and B. reaching care. Data were available for A. 1151 and B. 1042 patients. Respondantcould choose more than one reason for delay.

Appendix 5. Perceived reason for delay in seeking care in different (A) mechanisms and (B) Type of injury

Appendix 5A shows the perceived reasons for delay in seeking care based on mechanism of injury. Data were available for 1150 of 1151 patients eligible to answer thois question.

Appendix 5B shows shows the perceived reasons for delay in seeking care based on type of injury. Data were available for 1147 of 1151 patients eligible to answer thois question.

Appendix 6. Multivariable analysis of the association of having one hour delay with the demographic, injury related and health system variables in Ghana, Pakistan, Rwanda and South Africa

|  | **Ghana (n=1689)** | | | **Pakistan (n=1639)** | | | **Rwanda (n=1033)** | | | **South Africa (n=1447)** | | |
| --- | --- | --- | --- | --- | --- | --- | --- | --- | --- | --- | --- | --- |
|  | **AOR** | **95% CI** | **P value** | **AOR** | **95% CI** | **P value** | **AOR** | **95% CI** | **P value** | **AOR** | **95% CI** | **P value** |
| **Age** | 1.01 | 1-1.02 | 0.005 | 1.01 | 1-1.02 | 0.004 | 1.00 | 0.99-1.01 | 0.50 | 1.01 | 1-1.02 | 0.022 |
|  |  |  |  |  |  |  |  |  |  |  |  |  |
| **Sex**  (Ref: female) | 0.85 | 0.59-1.2 | 0.353 | 0.72 | 0.53-0.97 | 0.033 | 0.93 | 0.61-1.43 | 0.745 | 0.93 | 0.7-1.24 | 0.626 |
| **Education** (Ref: Secondary or higher) |  |  |  |  |  |  |  |  |  |  |  |  |
| No formal | 1.59 | 1.08-2.33 | 0.019 | 0.69 | 0.51-0.93 | 0.016 | 1.39 | 0.81-2.38 | 0.232 | 0.66 | 0.41-1.07 | 0.09 |
| Primary completed | 1.84 | 1.29-2.63 | 0.001 | 1.38 | 1.02-1.86 | 0.034 | 1.34 | 0.81-2.2 | 0.254 | 0.94 | 0.73-1.2 | 0.619 |
|  |  |  |  |  |  |  |  |  |  |  |  |  |
| **Wealth quintiles**  (Ref: Q5) |  |  |  |  |  |  |  |  |  |  |  |  |
| Q1 | 1.63 | 1.01-2.63 | 0.044 | 2.58 | 1.51-4.39 | <0.001 | 1.39 | 0.68-2.84 | 0.367 | 1.34 | 0.92-1.95 | 0.131 |
| Q2 | 1.40 | 0.88-2.23 | 0.159 | 3.01 | 2.02-4.47 | <0.001 | 1.16 | 0.64-2.1 | 0.633 | 1.07 | 0.73-1.55 | 0.735 |
| Q3 | 1.44 | 0.91-2.28 | 0.119 | 2.2 | 1.53-3.17 | <0.001 | 1.19 | 0.7-2.04 | 0.523 | 1.16 | 0.8-1.68 | 0.43 |
| Q4 | 0.77 | 0.49-1.21 | 0.259 | 1.63 | 1.15-2.3 | 0.006 | 1.38 | 0.85-2.25 | 0.194 | 0.75 | 0.52-1.09 | 0.13 |
|  |  |  |  |  |  |  |  |  |  |  |  |  |
| **Mechanism**  (Ref: RTC) |  |  |  |  |  |  |  |  |  |  |  |  |
| IPV | 1.26 | 0.71-2.22 | 0.436 | 0.75 | 0.5-1.11 | 0.153 | 1.21 | 0.54-2.7 | 0.643 | 1.12 | 0.8-1.58 | 0.514 |
| Fall | 2.05 | 1.29-3.25 | 0.002 | 0.87 | 0.64-1.18 | 0.364 | 1.06 | 0.66-1.7 | 0.816 | 1.53 | 1.05-2.24 | 0.026 |
| Hit | 1.14 | 0.53-2.47 | 0.730 | 1.27 | 0.57-2.84 | 0.552 | 1.5 | 0.61-3.69 | 0.374 | 0.89 | 0.36-2.2 | 0.793 |
| Fire or heat | 5.56 | 1.68-18.36 | 0.005 | 0.51 | 0.14-1.77 | 0.287 | 0.99 | 0.37-2.67 | 0.984 | 2.3 | 0.86-6.11 | 0.096 |
| Other | 3.89 | 2.12-7.14 | <0.001 | 0.68 | 0.33-1.39 | 0.289 | 1.31 | 0.66-2.62 | 0.442 | 1.18 | 0.69-2.03 | 0.546 |
|  |  |  |  |  |  |  |  |  |  |  |  |  |
| **Injury severity**  (Ref: Mild) |  |  |  |  |  |  |  |  |  |  |  |  |
| Moderate | 1.14 | 0.83-1.57 | 0.408 | 1.42 | 1.12-1.8 | 0.004 | 0.85 | 0.58-1.25 | 0.410 | 1.23 | 0.94-1.61 | 0.125 |
| Severe | 1.11 | 0.59-2.09 | 0.756 | 1.88 | 1.19-2.98 | 0.007 | 0.64 | 0.28-1.46 | 0.284 | 1.26 | 0.59-2.67 | 0.553 |
|  |  |  |  |  |  |  |  |  |  |  |  |  |
| **Prior healthcare encounter**  (Ref: no prior health encounter) | 29.65 | 21.44-40.99 | <0.001 | 5.49 | 4.38-6.89 | <0.001 | 12.31 | 8.43-17.99 | <0.001 | 3.7 | 2.9-4.73 | <0.001 |
|  |  |  |  |  |  |  |  |  |  |  |  |  |
| **Transport by ambulance**  (Ref: other) | 1.95 | 1.3-2.93 | 0.001 | 0.74 | 0.57-0.95 | 0.017 | 2.01 | 1.3-3.11 | 0.002 | 2.03 | 1.61-2.56 | <0.001 |
|  |  |  |  |  |  |  |  |  |  |  |  |  |
| **Urban catchment area**  (Ref: rural) | 2.35 | 1.07-5.16 | 0.034 | 1.82 | 1.23-2.69 | 0.003 | 0.36 | 0.2-0.63 | <0.001 | 1.6 | 1.19-2.16 | 0.002 |
|  |  |  |  |  |  |  |  |  |  |  |  |  |
| **Tertiary hospital**  (Ref: secondary) | 0.98 | 0.46-2.11 | 0.968 | 0.68 | 0.08-5.73 | 0.723 |  |  |  | 1.58 | 1.16-2.15 | 0.004 |

RCT: Road Traffic Collisions, IPV: Interpersonal Violence, AOR: adjusted odds ratio

Note: Hospital type omitted from Rwanda model for collinearity.

Appendix 7. Multivariable analysis showing association of experiencing delays to definitive care after moderate to severe injury using modified Poisson regression.

|  | **One-Hour Delay** | | | **Two-Hours Delay** | | |
| --- | --- | --- | --- | --- | --- | --- |
|  | **aPR** | **95% CI** | **P value** | **aPR** | **95% CI** | **P value** |
| **Age** | 1.00 | 1-1 | <0.001 | 1.01 | 1.01-1.01 | <0.001 |
|  |  |  |  |  |  |  |
| **Sex** (Ref: female) | 0.95 | 0.91-1 | 0.058 | 0.98 | 0.91-1.05 | 0.485 |
|  |  |  |  |  |  |  |
| **Education** (Ref: Secondary or higher) |  |  |  |  |  |  |
| No formal education | 1.01 | 0.95-1.07 | 0.779 | 1.12 | 1.03-1.21 | 0.006 |
| Primary completed | 1.08 | 1.03-1.14 | 0.002 | 1.10 | 1.02-1.19 | 0.012 |
|  |  |  |  |  |  |  |
| **Country** (Ref: South Africa) |  |  |  |  |  |  |
| Ghana | 1.12 | 1.04-1.21 | 0.003 | 1.29 | 1.15-1.44 | <0.001 |
| Pakistan | 0.93 | 0.86-1 | 0.061 | 1.06 | 0.94-1.19 | 0.337 |
| Rwanda | 1.30 | 1.21-1.4 | <0.001 | 1.51 | 1.36-1.68 | <0.001 |
|  |  |  |  |  |  |  |
| **Wealth quintiles** (Ref: Q5) |  |  |  |  |  |  |
| Q1 | 1.11 | 1.04-1.19 | 0.003 | 1.14 | 1.03-1.27 | 0.010 |
| Q2 | 1.12 | 1.05-1.2 | 0.001 | 1.13 | 1.03-1.25 | 0.012 |
| Q3 | 1.10 | 1.03-1.18 | 0.005 | 1.10 | 0.99-1.21 | 0.067 |
| Q4 | 1.00 | 0.93-1.07 | 0.970 | 1.01 | 0.92-1.12 | 0.794 |
|  |  |  |  |  |  |  |
| **Mechanism** (Ref: RTC*) |  | |  |  |  |  |
| Interpersonal Violence | 1.01 | 0.94-1.08 | 0.791 | 1.07 | 0.97-1.18 | 0.175 |
| Fall | 1.09 | 1.03-1.15 | 0.003 | 1.15 | 1.06-1.24 | 0.001 |
| Hit by a falling object | 1.10 | 1.01-1.19 | 0.031 | 1.27 | 1.13-1.43 | <0.001 |
| Fire or heat | 1.12 | 1-1.26 | 0.044 | 1.18 | 0.98-1.41 | 0.076 |
| Other | 1.10 | 1.02-1.19 | 0.012 | 1.13 | 1.01-1.26 | 0.031 |
|  |  |  |  |  |  |  |
| **Injury severity** (Ref: Mild) |  |  |  |  |  |  |
| Moderate | 1.08 | 1.03-1.13 | 0.001 | 1.00 | 0.94-1.07 | 0.993 |
| Severe | 1.11 | 1.01-1.21 | 0.032 | 0.85 | 0.72-0.99 | 0.043 |
|  |  |  |  |  |  |  |
| **Prior healthcare encounter** (Ref: no prior health encounter) | 2.80 | 2.61-3.02 | <0.001 | 5.89 | 5.15-6.73 | <0.001 |
|  |  |  |  |  |  |  |
| **Transport by ambulance** (Ref: other modes of transport) | 1.11 | 1.06-1.16 | <0.001 | 1.09 | 1.02-1.16 | 0.013 |
|  |  |  |  |  |  |  |
| **Urban catchment area** (Ref: rural) | 1.16 | 1.07-1.26 | 0.001 | 1.20 | 1.06-1.36 | 0.003 |
|  |  |  |  |  |  |  |
| **Tertiary hospital** (Ref: secondary) | 1.10 | 1-1.21 | 0.057 | 1.08 | 0.94-1.23 | 0.292 |
|  |  |  |  |  |  |  |

aPR: adjusted prevalence ratio,

5808 complete cases were included.

Appendix 8. Multivariable logistic analysis of the association of experiencing one hour delay with the demographic, injury related, and health system variables, A. with imputation, B. with imputation and interaction between country and ambulance transport, and C. in the complete-case dataset with interaction between country and ambulance transport.

|  | **A. One-Hour Delay, (MI)** | | | **B. One-hour Delay with Interaction (MI)** | | | **C. One-hour Delay with Interaction (complete case)** | | | |
| --- | --- | --- | --- | --- | --- | --- | --- | --- | --- | --- |
|  | **AOR** | **95% CI** | **P value** | **AOR** | **95% CI** | **P value** | **AOR** | **95% CI** | **P value** | |
| **Age** | 1.00 | 1-1.01 | 0.016 | 1.00 | 1.00-1.01 | 0.002 | 1.01 | 1.01-1.01 | | <0.001 |
|  |  |  |  |  |  |  |  |  | |  |
| **Sex** (Ref: female) | 0.88 | 0.78-1 | 0.044 | 0.92 | 0.81-1.04 | 0.119 | 0.89 | 0.76-1.04 | | 0.148 |
|  |  |  |  |  |  |  |  |  | |  |
| **Education** (Ref: Secondary or higher) |  |  |  |  |  |  |  |  | |  |
| No formal education | 1.08 | 0.94-1.23 | 0.265 | 1.09 | 0.95-1.24 | 0.233 | 1.01 | 0.86-1.2 | | 0.875 |
| Primary completed | 1.71 | 1.52-1.93 | <0.001 | 1.61 | 1.42-1.83 | <0.001 | 1.29 | 1.11-1.5 | | 0.001 |
|  |  |  |  |  |  |  |  |  | |  |
| **Wealth quintiles** (Ref: Q5) |  |  |  |  |  |  |  |  | |  |
| Q1 | 1.26 | 1.06-1.5 | 0.010 | 1.25 | 1.04-1.5 | 0.016 | 1.56 | 1.26-1.93 | | <0.001 |
| Q2 | 1.45 | 1.22-1.72 | <0.001 | 1.44 | 1.21-1.71 | <0.001 | 1.57 | 1.28-1.93 | | <0.001 |
| Q3 | 1.36 | 1.15-1.61 | <0.001 | 1.41 | 1.19-1.69 | <0.001 | 1.48 | 1.22-1.81 | | <0.001 |
| Q4 | 1.08 | 0.91-1.27 | 0.366 | 1.08 | 0.91-1.28 | 0.372 | 1.04 | 0.85-1.26 | | 0.720 |
|  |  |  |  |  |  |  |  |  | |  |
| **Mechanism** (Ref: RTC*) |  | |  |  |  |  |  |  | |  |
| Interpersonal Violence | 1.01 | 0.86-1.18 | 0.895 | 0.95 | 0.81-1.11 | 0.527 | 1.06 | 0.86-1.29 | | 0.597 |
| Fall | 1.15 | 1-1.33 | 0.045 | 1.12 | 0.97-1.29 | 0.128 | 1.32 | 1.11-1.59 | | 0.002 |
| Hit by a falling object | 1.24 | 0.93-1.66 | 0.138 | 1.28 | 0.95-1.71 | 0.101 | 1.30 | 0.89-1.9 | | 0.169 |
| Fire or heat | 1.14 | 0.84-1.54 | 0.412 | 1.15 | 0.84-1.57 | 0.387 | 1.62 | 0.98-2.69 | | 0.062 |
| Other | 1.60 | 1.28-2.01 | <0.001 | 1.55 | 1.24-1.95 | <0.001 | 1.44 | 1.07-1.92 | | 0.014 |
|  |  |  |  |  |  |  |  |  | |  |
| **Injury severity** (Ref: Mild) |  |  |  |  |  |  |  |  | |  |
| Moderate | 1.34 | 1.18-1.52 | <0.001 | 1.31 | 1.16-1.48 | <0.001 | 1.26 | 1.1-1.45 | | 0.001 |
| Severe | 1.28 | 0.97-1.68 | 0.086 | 1.22 | 0.92-1.62 | 0.173 | 1.32 | 0.98-1.78 | | 0.068 |
|  |  |  |  |  |  |  |  |  | |  |
| **Prior healthcare encounter** (Ref: no prior health encounter) | 6.77 | 6.05-7.59 | <0.001 | 6.94 | 6.19-7.79 | <0.001 | 8.25 | 7.23-9.41 | | <0.001 |
|  |  |  |  |  |  |  |  |  | |  |
| **Transport by ambulance** (Ref: other modes of transport) | 0.90 | 0.81-1.01 | 0.075 | 1.97 | 1.59-2.44 | <0.001 | 1.92 | 1.51-2.44 | | <0.001 |
|  |  |  |  |  |  |  |  |  | |  |
| **Country** (Ref: South Africa) |  |  |  |  |  |  |  |  | |  |
| Ghana | 1.70 | 1.43-2.03 | <0.001 | 1.79 | 1.45-2.2 | <0.001 | 1.65 | 1.28-2.12 | | <0.001 |
| Pakistan | 2.07 | 1.76-2.44 | <0.001 | 6.27 | 5.01-7.84 | <0.001 | 1.52 | 1.14-2.03 | | 0.004 |
| Rwanda | 2.33 | 1.96-2.77 | <0.001 | 2.75 | 2.15-3.52 | <0.001 | 4.22 | 2.96-6.01 | | <0.001 |
|  |  |  |  |  |  |  |  |  | |  |
| **Interaction (transport by ambulance * country)** |  |  |  |  |  |  |  |  | |  |
| Ambulance in Ghana |  |  |  | 1.48 | 1.04-2.11 | 0.031 | 1.34 | 0.89-2 | | 0.159 |
| Ambulance in Pakistan |  |  |  | 0.12 | 0.09-0.16 | <0.001 | 0.41 | 0.29-0.57 | | <0.001 |
| Ambulance in Rwanda |  |  |  | 0.54 | 0.4-0.75 | <0.001 | 0.56 | 0.36-0.86 | | 0.008 |
|  |  |  |  |  |  |  |  |  | |  |
| **Urban catchment area**  (Ref: rural) | 0.41 | 0.35-0.48 | <0.001 | 0.58 | 0.49-0.68 | <0.001 | 1.48 | 1.21-1.8 | | <0.001 |
|  |  |  |  |  |  |  |  |  | |  |
| **Tertiary hospital**  (Ref: secondary) | 2.89 | 2.4-3.48 | <0.001 | 2.02 | 1.67-2.46 | <0.001 | 1.29 | 1.03-1.63 | | 0.030 |

Considering the interaction between countries and ambulances after imputation (B), the adjusted odds of delay among patients arriving by ambulance compared with those who did not were:

- Ghana: 2.91 (95%CI: 1.92 – 4.41), p value < 0.001
- Pakistan: 0.24 (95%CI: 0.17 – 0.34), p value < 0.001
- Rwanda: 1.07 (95%CI: 0.73 – 1.57), p value: 0.74
- South Africa (reference group): 1.97 (95%CI: 1.59-2.44), p value< 0.001

Considering the interaction between countries and ambulances in the complete case dataset (C), the adjusted odds of delay among patients arriving by ambulance compared with those who did not were:

- Ghana: 2.57 (95%CI: 1.85-3.55), p value< 0.001
- Pakistan: 0.78 (95%CI: 0.61-1.00), p value: 0.049
- Rwanda: 1.07 (95%CI: 0.75-1.53), p value: 0.717
- South Africa (reference group): 1.92 (95%CI: 1.51-2.44), p value< 0.001

Appendix 9. Multivariable analysis of the association of experiencing one hour delay with the demographic, injury related and health system variables A. after removing catchment area from the model and B. after adding injury type to the model

|  | **A. Not including catchment variable** | | | **B. Adding injury type to the main model** | | |
| --- | --- | --- | --- | --- | --- | --- |
|  | **AOR** | **95% CI** | **P value** | **AOR** | **95% CI** | **P value** |
| **Age** | 1.01 | 1-1.01 | <0.001 | 1.01 | 1-1.01 | <0.001 |
|  |  |  |  |  |  |  |
| **Sex** (Ref: female) | 0.87 | 0.75-1.02 | 0.087 | 0.86 | 0.73-1.00 | 0.055 |
|  |  |  |  |  |  |  |
| **Education** (Ref: Secondary or higher) |  |  |  |  |  |  |
| No formal education | 1.00 | 0.85-1.19 | 0.961 | 1.05 | 0.89-1.25 | 0.565 |
| Primary completed | 1.27 | 1.10-1.48 | 0.001 | 1.29 | 1.11-1.50 | 0.001 |
|  |  |  |  |  |  |  |
| **Country** (Ref: South Africa) |  |  |  |  |  |  |
| Ghana | 1.85 | 1.51-2.27 | <0.001 | 1.52 | 1.22-1.89 | <0.001 |
| Pakistan | 0.92 | 0.75-1.12 | 0.387 | 0.83 | 0.68-1.02 | 0.077 |
| Rwanda | 3.42 | 2.72-4.30 | <0.001 | 2.98 | 2.35-3.79 | <0.001 |
|  |  |  |  |  |  |  |
| **Wealth quintiles** (Ref: Q5) |  |  |  |  |  |  |
| Q1 | 1.37 | 1.11-1.68 | 0.003 | 1.51 | 1.22-1.89 | <0.001 |
| Q2 | 1.48 | 1.21-1.81 | <0.001 | 1.52 | 1.24-1.87 | <0.001 |
| Q3 | 1.40 | 1.15-1.70 | 0.001 | 1.40 | 1.15-1.70 | 0.001 |
| Q4 | 1.02 | 0.84-1.24 | 0.825 | 1.02 | 0.84-1.23 | 0.875 |
|  |  |  |  |  |  |  |
| **Mechanism** (Ref: RTC*) |  | |  |  |  |  |
| Interpersonal Violence | 1.14 | 0.94-1.39 | 0.196 | 1.16 | 0.94-1.43 | 0.172 |
| Fall | 1.42 | 1.19-1.70 | <0.001 | 1.45 | 1.21-1.74 | <0.001 |
| Hit by a falling object | 1.36 | 0.93-1.98 | 0.111 | 1.42 | 0.97-2.07 | 0.071 |
| Fire or heat | 1.68 | 1.02-2.77 | 0.043 | 1.99 | 1.16-3.43 | 0.013 |
| Other | 1.49 | 1.12-1.99 | 0.007 | 1.61 | 1.20-2.17 | 0.002 |
|  |  |  |  |  |  |  |
| **Injury severity** (Ref: Mild) |  |  |  |  |  |  |
| Moderate | 1.30 | 1.13-1.49 | <0.001 | 1.11 | 0.94-1.32 | 0.214 |
| Severe | 1.41 | 1.05-1.89 | 0.024 | 1.17 | 1.84-1.62 | 0.349 |
|  |  |  |  |  |  |  |
| **Prior healthcare encounter** (Ref: no prior health encounter) | 8.53 | 7.49-9.71 | <0.001 | 8.65 | 7.59-9.87 | <0.001 |
|  |  |  |  |  |  |  |
| **Transport by ambulance** (Ref: other modes of transport) | 1.42 | 1.23-1.63 | <0.001 | 1.38 | 1.20-1.59 | <0.001 |
|  |  |  |  |  |  |  |
| **Urban catchment area**  (Ref: rural) |  |  |  | 1.41 | 1.16-1.73 | 0.001 |
|  |  |  |  |  |  |  |
| **Tertiary hospital**  (Ref: secondary) | 1.77 | 1.49-2.09 | <0.001 | 1.30 | 1.03-1.64 | 0.027 |
|  |  |  |  |  |  |  |
| **Injury type (Ref:** Neurotrauma |  |  |  |  |  |  |
| Bone or joint |  |  |  | 0.60 | 0.49-0.73 | <0.001 |
| Cuts or soft tissue |  |  |  | 0.50 | 0.38-0.65 | <0.001 |
| Other isolated injuries |  |  |  | 0.57 | 0.42-0.77 | <0.001 |
| Polytrauma |  |  |  | 0.77 | 0.61-0.97 | 0.027 |

* AOR: adjusted odds ratio

- 5808 complete cases were included.

Appendix 10. Multivariable analysis of the association of experiencing one hour delay with the demographic, injury related and health system variables, A. with interaction term of transport by ambulance and prior healthcare encounter. B. in those not attending any previous facility.

|  | **A.One-Hour Delay** | | | **B. Not including those who have attended a prior facility** | | |
| --- | --- | --- | --- | --- | --- | --- |
|  | **AOR** | **95% CI** | **P value** | **AOR** | **95% CI** | **P value** |
| **Age** | 1.01 | 1-1.01 | <0.001 | 1.01 | 1-1.01 | 0.017 |
|  |  |  |  |  |  |  |
| **Sex** (Ref: female) | 0.87 | 0.74-1.01 | 0.073 | 0.84 | 0.67-1.06 | 0.153 |
|  |  |  |  |  |  |  |
| **Education** (Ref: Secondary or higher) |  |  |  |  |  |  |
| No formal education | 1.01 | 0.86-1.2 | 0.866 | 1.11 | 0.86-1.43 | 0.439 |
| Primary completed | 1.26 | 1.09-1.47 | 0.002 | 1.44 | 1.16-1.8 | 0.001 |
|  |  |  |  |  |  |  |
| **Country** (Ref: South Africa) |  |  |  |  |  |  |
| Ghana | 1.74 | 1.39-2.17 | <0.001 | 0.43 | 0.3-0.61 | <0.001 |
| Pakistan | 0.88 | 0.72-1.07 | 0.200 | 0.69 | 0.51-0.94 | 0.017 |
| Rwanda | 3.04 | 2.4-3.86 | <0.001 | 1.19 | 0.81-1.74 | 0.371 |
|  |  |  |  |  |  |  |
| **Wealth quintiles** (Ref: Q5) |  |  |  |  |  |  |
| Q1 | 1.53 | 1.23-1.9 | <0.001 | 1.34 | 0.95-1.89 | 0.09 |
| Q2 | 1.57 | 1.28-1.92 | <0.001 | 1.53 | 1.12-2.09 | 0.007 |
| Q3 | 1.44 | 1.18-1.75 | <0.001 | 1.43 | 1.06-1.92 | 0.017 |
| Q4 | 1.03 | 0.85-1.25 | 0.754 | 1.06 | 0.8-1.4 | 0.709 |
|  |  |  |  |  |  |  |
| **Mechanism** (Ref: RTC*) |  | |  |  |  |  |
| Interpersonal Violence | 1.1 | 0.9-1.35 | 0.33 | 1.08 | 0.78-1.48 | 0.655 |
| Fall | 1.41 | 1.18-1.69 | <0.001 | 1.28 | 0.98-1.66 | 0.071 |
| Hit by a falling object | 1.34 | 0.92-1.96 | 0.132 | 0.6 | 0.27-1.31 | 0.197 |
| Fire or heat | 1.80 | 1.08-3.01 | 0.025 | 1.19 | 0.52-2.72 | 0.675 |
| Other | 1.46 | 1.09-1.95 | 0.011 | 1.16 | 0.73-1.85 | 0.537 |
|  |  |  |  |  |  |  |
| **Injury severity** (Ref: Mild) |  |  |  |  |  |  |
| Moderate | 1.28 | 1.11-1.47 | 0.001 | 1.27 | 1.03-1.56 | 0.024 |
| Severe | 1.36 | 1.01-1.82 | 0.043 | 1.30 | 0.85-1.99 | 0.223 |
|  |  |  |  |  |  |  |
| **Prior healthcare encounter** (Ref: no prior health encounter) | 10.83 | 8.97-13.08 | <0.001 |  |  |  |
|  |  |  |  |  |  |  |
| **Transport by ambulance** (Ref: other modes of transport) | 1.9 | 1.54-2.36 | <0.001 | 1.28 | 1.02-1.62 | 0.034 |
|  |  |  |  |  |  |  |
| **The interaction term of ambulance and prior healthcare encounter** | 0.61 | 0.47-0.79 | <0.001 |  |  |  |
|  |  |  |  |  |  |  |
| **Urban catchment area**  (Ref: rural) | 1.38 | 1.13-1.69 | 0.002 | 2.13 | 1.57-2.88 | <0.001 |
|  |  |  |  |  |  |  |
| **Tertiary hospital**  (Ref: secondary) | 1.33 | 1.06-1.68 | 0.015 | 0.70 | 0.5-0.99 | 0.045 |

RCT: Road Traffic Collisions, IPV: Interpersonal Violence, AOR: adjusted odds ratio. Number of Included cases was A. 5808 and B. 2585

The odds ratio for delays in those who visited a prior facility and were transported by ambulance (compared to those arrived directly to the definitive care facility with other means of transport is 12.61 (95% CI: 10.47-15.18).
